# Walking the interactome to identify human miRNA-disease associations through the functional link between miRNA targets and disease genes

**DOI:** 10.1186/1752-0509-7-101

**Published:** 2013-10-08

**Authors:** Hongbo Shi, Juan Xu, Guangde Zhang, Liangde Xu, Chunquan Li, Li Wang, Zheng Zhao, Wei Jiang, Zheng Guo, Xia Li

**Affiliations:** 1 College of Bioinformatics Science and Technology and State-Province Key Laboratories of Biomedicine-Pharmaceutics of China, Harbin Medical University, Harbin, Heilongjiang 150081, PR China; 2 Department of Cardiology, The Fourth Affiliated Hospital of Harbin Medical University, Harbin, Heilongjiang 150001, PR China

**Keywords:** MiRNA, Disease genes, Random walk analysis, MiRNA-disease network

## Abstract

**Background:**

MicroRNAs (miRNAs) are important post-transcriptional regulators that have been demonstrated to play an important role in human diseases. Elucidating the associations between miRNAs and diseases at the systematic level will deepen our understanding of the molecular mechanisms of diseases. However, miRNA-disease associations identified by previous computational methods are far from completeness and more effort is needed.

**Results:**

We developed a computational framework to identify miRNA-disease associations by performing random walk analysis, and focused on the functional link between miRNA targets and disease genes in protein-protein interaction (PPI) networks. Furthermore, a bipartite miRNA-disease network was constructed, from which several miRNA-disease co-regulated modules were identified by hierarchical clustering analysis. Our approach achieved satisfactory performance in identifying known cancer-related miRNAs for nine human cancers with an area under the ROC curve (AUC) ranging from 71.3% to 91.3%. By systematically analyzing the global properties of the miRNA-disease network, we found that only a small number of miRNAs regulated genes involved in various diseases, genes associated with neurological diseases were preferentially regulated by miRNAs and some immunological diseases were associated with several specific miRNAs. We also observed that most diseases in the same co-regulated module tended to belong to the same disease category, indicating that these diseases might share similar miRNA regulatory mechanisms.

**Conclusions:**

In this study, we present a computational framework to identify miRNA-disease associations, and further construct a bipartite miRNA-disease network for systematically analyzing the global properties of miRNA regulation of disease genes. Our findings provide a broad perspective on the relationships between miRNAs and diseases and could potentially aid future research efforts concerning miRNA involvement in disease pathogenesis.

## Background

MicroRNAs (MiRNAs) are important regulators that can strongly affect cellular functions including proliferation, differentiation, and apoptosis through post-transcriptional negative regulation of target gene expression [1]. Dysregulated expression of miRNAs has been previously demonstrated in human diseases, and there is a growing body of evidence regarding the important roles of miRNAs in human diseases [2]. Identification of disease-related miRNAs will aid in the pathological classification of diseases and help to formulate individualized treatment regimes [3].

Thus far, computational prediction methods for miRNA-disease associations have produced some valuable results. Under the assumption that functionally related miRNAs tend to be associated with phenotypically similar diseases [4], Jiang et al. [5] used a hypergeometric distribution to construct a miRNA functional network and used phenotype similarity information to infer potential miRNA-disease associations. The hypergeometric distribution method considers the number of overlapping genes while neglecting the functional link between them, and the scoring system used in their study only considered the direct neighbour information of each miRNA in the miRNA functional network. Chen et al. [6] assessed potential miRNA-disease interactions through a miRNA-miRNA functional similarity network that was constructed based on the similarity of miRNA-associated diseases. However, this method is not applicable to diseases that have no known related miRNAs.

MiRNA mainly performs its regulatory function through its targets, and thus we presumed that if targets of a miRNA correlate with disease genes then the miRNA tends to be associated with the disease. Functional connections between miRNA targets and disease genes could be obtained via PPI network. Functional PPI networks include information on physical interactions, functional communication, and associations between the expression levels of genes, and they serve as an important foundation for understanding the functional roles of biomolecules [7,8]. In addition, random walk analysis is a global network distance measurement that is usually used to measure similarities between the nodes of a network, and previous reports have demonstrated its effectiveness in candidate disease gene prioritization [9,10]. Random walk analysis has been shown to outperform many existing local network-based gene prioritization algorithms [9,10]. Therefore, we proposed a new algorithm for identifying miRNA-disease associations.

Additionally, dissection of miRNA-disease networks can reveal regulatory mechanisms of human diseases from different perspectives. Currently, a miRNA-disease network can be constructed primarily using three different methods. The first method is based on published report mining. For example, Lu et al. [4] built a human miRNA-disease bipartite network by manually collecting miRNA-disease association data from publications. This method generally includes only a few types of interactions, thus causing a lack of systematization [11]. The second approach involves applying unbiased high-throughput experiments to the whole miRNAome. Although current technological progress suggests that comprehensive human biological network maps will be completed in the next few years, this method remains difficult to initiate [12]. The third method involves computational prediction that can quickly and effectively predict miRNA-disease associations to construct a miRNA-disease network. Such a network generally contains large numbers of nodes and edges to meet the needs of systematic analysis.

In this study, we developed a computational framework to identify potential miRNA-disease associations by taking advantage of the functional connections between miRNA targets and disease genes in protein-protein interaction (PPI) networks. The predicted miRNA-disease associations were provided to identify novel miRNAs with aberrant expression in human diseases. Furthermore, we constructed a miRNA-disease network and analyzed its features, and found that some miRNAs combined to regulate disease-related genes in the same disease class.

## Methods

### Human protein-protein interaction (PPI) data and random PPI networks

The PPI data for human was compiled from the Human Protein Reference Database (HPRD Release 9) containing annotations pertaining to human proteins based on experimental evidence from published reports [13]. The entire network contained 9453 genes and 36867 interactions. We mapped gene names to Entrez gene IDs and then obtained the maximum components of the whole network, which contains 9028 genes and 35865 interactions. It is noteworthy that PPI data in HPRD were annotated as common to all protein isoforms, primarily because of the general lack of experimental data [13]. A total of 1,000 random PPI networks were acquired by randomly shuffling the above PPI network while maintaining the degree of each node unchanged.

### Disease genes and miRNA targets

The disease-gene association data were obtained from a study by Li [14], which contained 15149 relationships involving 412 diseases and 2831 disease genes that belong to 18 disease classes. MiRNA target genes were acquired from seven miRNA target databases: miRanda [15], PicTar [16], TargetScan [17], DIANA-microT [18], RNA22 [19], RNAhybrid [20], and miRBase Targets [21]. We extracted the regulatory associations between miRNAs and targets, which appeared in at least three databases in order to increase the reliability of the results. In total, we obtained 52828 targeting pairs that involved 566 miRNAs and 8085 target genes. This method has also been adopted in a previous study [22]. After the above disease genes and miRNA targets were annotated to the HPRD network, 269 diseases and 499 miRNAs with target genes more than five were remained, including 2160 disease genes.

### Identification of miRNA-disease pairs and construction of a miRNA-disease network

MiRNA mainly performs its regulatory function through its targets. We thus presumed that if targets of a miRNA are correlated with disease genes, the miRNA tends to be associated with the disease. Based on this hypothesis, we used a framework to identify miRNA-disease associations and further constructed a miRNA-disease network.

**Figure 1:**
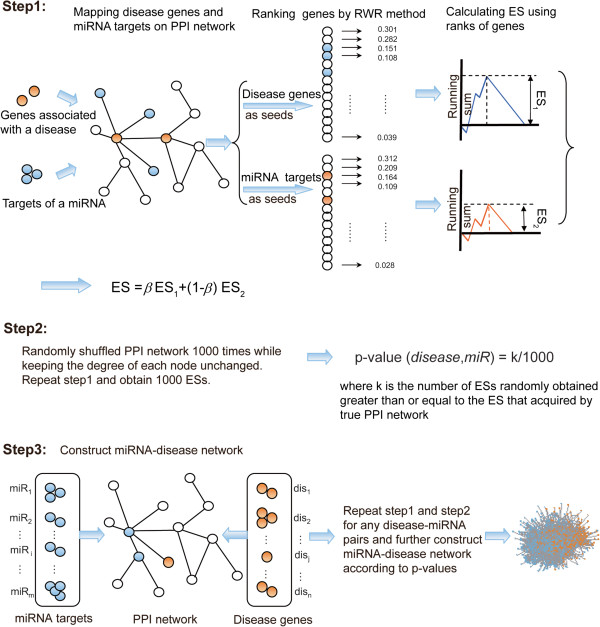
**An overview of the construction of the miRNA-disease network.** Step 1: For a given miRNA and disease, we used random walk analysis using the disease genes as seeds and the miRNA targets as seeds simultaneously to obtain the ES. Step 2: Computation of p-value, used to measure the potential regulatory relationship between the miRNA and disease. Step 3: We repeated step 1 and step 2 for any disease-miRNA pair and further adopted all of the significant miRNA-disease pairs to construct a miRNA-disease network.

The strategy to identify miRNA-disease pairs using our model is shown in Figure 1. For a miRNA-disease pair, firstly, we mapped the causal genes of the disease and the miRNA target genes onto the PPI network. Then, we obtained a gene rank list using the random walk with restart (RWR) algorithm (see Additional file 1) with the disease genes serving as seeds. Every miRNA target gene was given a probability value in the above ranked gene list. The larger the probability value, the more similar the miRNA target gene was to the known disease gene. The miRNA targets that ranked at the top of the list should exhibit a stronger association with the disease, because these targets have a higher similarity to disease genes compared with those ranked at the bottom of the list. The ranked gene list used in this study was obtained using the RWR algorithm with disease genes as seeds, derived from gene set enrichment analysis (GSEA) [23], We defined ES_1_ (enrichment score) using the following formula:

(1) $${\text{ES}}_{1}=\max_{1\leq i\leq N}\sum_{\begin{array}{l} g_{j}\in \text{TG}\\ j\leq i \end{array}}\sqrt{\left(N-n_{1}\right)/n_{1}}-\sum_{\begin{array}{l} g_{j}\notin \text{TG}\\ j\leq i \end{array}}\sqrt{n_{1}/\left(N-n_{1}\right)\;)}$$

where $\text{TG}=\left\{g_{1},g_{2},\ldots ,g_{n_{1}}\right\}$ denotes the miRNA target gene set including *n*_1_ genes. The gene rank list *L* = {*g*_1_, *g*_2_, …, *g*_*N*_} obtained included *N* genes, where *N* represents the number of genes involved in the PPI network. The miRNA targets $\text{TG}=\left\{g_{1},g_{2},\ldots ,g_{n_{1}}\right\}$ were ranked in this gene list. Subsequently, we calculated a running sum statistic. Beginning with the top-ranking gene, the running sum was calculated by walking down the list with the running sum statistic incrementing by $\sqrt{\left(N-n_{1}\right)/n_{1}}$ to encounter a gene in *TG* and decrementing by $\sqrt{n_{1}/\left(N-n_{1}\right)}$ if the gene is not in *TG*. ES_1_ is defined as the greatest positive deviation of the running sum across all *N* genes. Similarly, for the same miRNA-disease pair referred to above, we computed ES_2_ by the RWR algorithm with miRNA target genes as seeds:

(2) $${\text{ES}}_{2}=\max_{1\leq i\leq N}\sum_{\begin{array}{l} g_{j}\in \text{DG}\\ j\leq i \end{array}}{\sqrt{\left(N-n_{2}\right)/n}}_{2}-\sum_{\begin{array}{l} g_{j}\notin \text{DG}\\ j\leq i \end{array}}\sqrt{n_{2}/\left(N-n_{2}\right)\;)}$$

where $\text{DG}=\left\{g_{1},g_{2},\ldots ,g_{n_{2}}\right\}$ denotes the disease gene set including *n*_2_ disease genes. Following the above procedure for the same miRNA-disease pair, we computed ES_1_ and ES_2_ using the RWR algorithm with disease genes as seeds and miRNA target genes as seeds, respectively. We then computed their combination as ES with the following formula:

(3) $$\text{ES}=\beta {\text{ES}}_{1}+\left(1-\beta \right){\text{ES}}_{2}$$

The parameter *β* ∈ (0, 1) is used to control the effect of two kinds of seed nodes, disease genes and miRNA targets. If *β* is 0.5, the seed nodes of disease genes and miRNA targets are weighted equally. If *β* is above 0.5, the seed nodes of disease genes are given more importance. In this study, we set *β* as 0.5.

Secondly, we used a p-value to measure the significance of the association between the miRNA and the disease. The p-value was defined as the fraction of randomly achieved ESs greater than or equal to the true ES. As stringent controls, 1000 random networks were constructed by preserving the number of direct neighbors for each protein in the original PPI network using the edge switching method [22,24-26]. This procedure enabled us to obtain 1,000 ESs while maintaining the network structure. The p-value was computed using the formula below:

(4) $$p‒\text{value}\;\left(\text{disease},\text{miR}\right)=k/1000$$

where *k* is the number of ESs computed by random PPI networks greater than or equal to the ES computed by the true PPI network. The p-value (*disease*, *miR*) reflects the correlation between the miRNA and the disease. The lower the p-value (*disease*, *miR*), the greater the probability that the miRNA is associated with the development, diagnosis, and prognosis of the disease.

Finally, we computed p-values for disease-miRNA pairs between 269 diseases and 499 miRNAs by applying the procedures described above. We set up a p-value threshold (e.g., 0.05) to determine whether a miRNA and a disease had a link. MiRNA and disease pairs with p-values less than the threshold will be connected by a direct link. Otherwise, they are not connected directly. Thus, a miRNA-disease network can be constructed using this approach. It is worth noting that for each disease, different p-value thresholds only affect the number of miRNA-disease associations, but not the rank of the miRNAs.

## Results

### Stable performance of our algorithm

To evaluate the performance of our algorithm in identifying miRNA-disease associations, we performed a validation on nine human cancers. The testing set for the performance of our method was selected as follows. For each cancer, the known cancer related miRNAs were obtained from miR2Disease [27] and HMDD [4] databases that provide a comprehensive record of miRNA deregulation involved in human diseases. We extracted the miRNA-cancer associations yielded by low-throughput methods such as northern blot and quantitative RT–PCR approaches as positive samples. In total, we obtained 518 known miRNA-cancer associations. The number of miRNAs associated with each cancer was different, ranging from nine to 104 (Additional file 1: Table S1). At present, collecting non-cancer related miRNA is difficult or even impossible. In this study, we chose miRNAs that exhibited the lowest fold change values as negative controls by analyzing the corresponding expression profile of the respective cancer. We also used the same number of negative controls as that of positive samples (Additional file 1: Table S1). MiRNA expression profiles of nine human cancers were downloaded from the Gene Expression Omnibus (GEO) and The Cancer Genome Atlas (TCGA) (for a detailed description, see Additional file 1). We scored miRNAs for each of the nine cancers according to our method. The score was then compared with a specified threshold *δ* with lower thresholds yielding more conservative predictions. True positives (TP) are miRNA-disease associations for known disease miRNAs below the threshold whereas false positives (FP) are associations that satisfy the p-value (*disease, miR*) ≤ *δ* but are not confirmed by current knowledge. True negatives (TN) are miRNA-disease associations that satisfy the p-value (*disease, miR*) ≤ *δ* for which the miRNAs are not currently known to be associated with the disease, whereas false negatives (FN) are miRNA-disease associations that correspond to known disease miRNAs but are above the threshold. The sensitivity is TP/(TP + FN), and the specificity is TN/(TN + FP). The ROC curve was plotted by computing the sensitivity and specificity while varying the threshold. At the same time, we calculated the corresponding area under the ROC curve (AUC) values for each cancer. The results are shown in Additional file 1: Table S2. AUC values ranged from 71.3 to 91.3% in all nine cancers, and the AUC values of three cancers exceeded 0.8. In addition, we computed the AUC value for all of the known 518 miRNA-cancer pairs together to evaluate the method, and we obtained an AUC value of 76.7%. These results indicated that our algorithm was effective for identification of miRNA-disease associations.

To evaluate the robustness of our method, we considered different networks, disease-related genes, and parameters. Signaling networks are a critical cell communication platform for disease development, In particular, strong evidence shows that cancer is a disease with abnormal cell signaling [28]. We implemented our method in a human signaling network that contains ~6,300 proteins and ~63,000 signaling relations [29-32]. As a result, the AUC values of nine cancers were comparable with that of the PPI network (Additional file 1: Table S3). Disease-related genes identified by DNA sequencing technology were also used to evaluate the robustness of our algorithm. Because of the lack of data, we assessed four kinds of cancer-related genes from published reports (breast cancer [33], glioma [34], ovarian cancer [35], and sarcoma [36]). The results showed that the AUC values of four cancers were slightly lower than that we obtained previously (Additional file 1: Table S4). In the first step of our algorithm, there is one parameter *β*, to investigate the stability of the algorithm, and we applied it to nine human cancers with a *β* range of 0.1 to 0.9 in increments of 0.1. The results are shown in Additional file 1: Table S5 and Figure S1. For each cancer, the AUC values did not change significantly as *β* varied. We also evaluated the effect of the restart probability *α* in the RWR algorithm. We set various values of *α* ranging from 0.1 to 0.9 with a step of 0.2. The AUC values for each cancer were calculated and results are shown in Additional file 1: Table S6. We found that, when this parameter ranged from 0.5 to 0.9, the performance became stable and performed slightly better. Thus, the dependence of our method on this parameter is slight, especially when the value of *α* is above 0.5. In addition, we observed that our algorithm was robust in 5000 random tests (Additional file 1: Table S7).

### Comparison with the existing methods

We compared our method with some existing methods. At present, several computational methods for miRNA-disease association prediction have been proposed based on different data sources, which makes it difficult to carry out comparisons. Jiang et al. [5] used hypergeometric distribution to construct a miRNA functional network for predicting miRNA-disease associations, and achieved an AUC value of 75.80%. In our study, we used a systematic approach to identify miRNA-disease associations, which was based on functional connections between miRNA targets and disease genes in PPI network, and a global network measure distance measure realized by RWR algorithm was utilized. By applying this method to nine human cancers, we achieved AUC values ranging from 71.3 to 91.3%. Chen et al. proposed a computational method to infer miRNA-disease associations based on random walk on the miRNA-miRNA functional network [6]. Although this method achieved a better AUC value of 86.17%, it was not applicable to diseases which have no known related miRNAs. In addition, the miRNA-miRNA functional similarity network they used was constructed previously, which included 271 miRNAs and the giant network component only contained 64 miRNAs. We also compared our method with the hypergeometric distribution method. A hypergeometric distribution was performed to measure the association of a miRNA and a disease by testing whether the overlap between miRNA targets and disease genes was statistically significant. The results showed that our strategy was more advantageous than the hypergeometric distribution method (Additional file 1: Table S8).

### Construction of a miRNA-disease network

**Figure 2:**
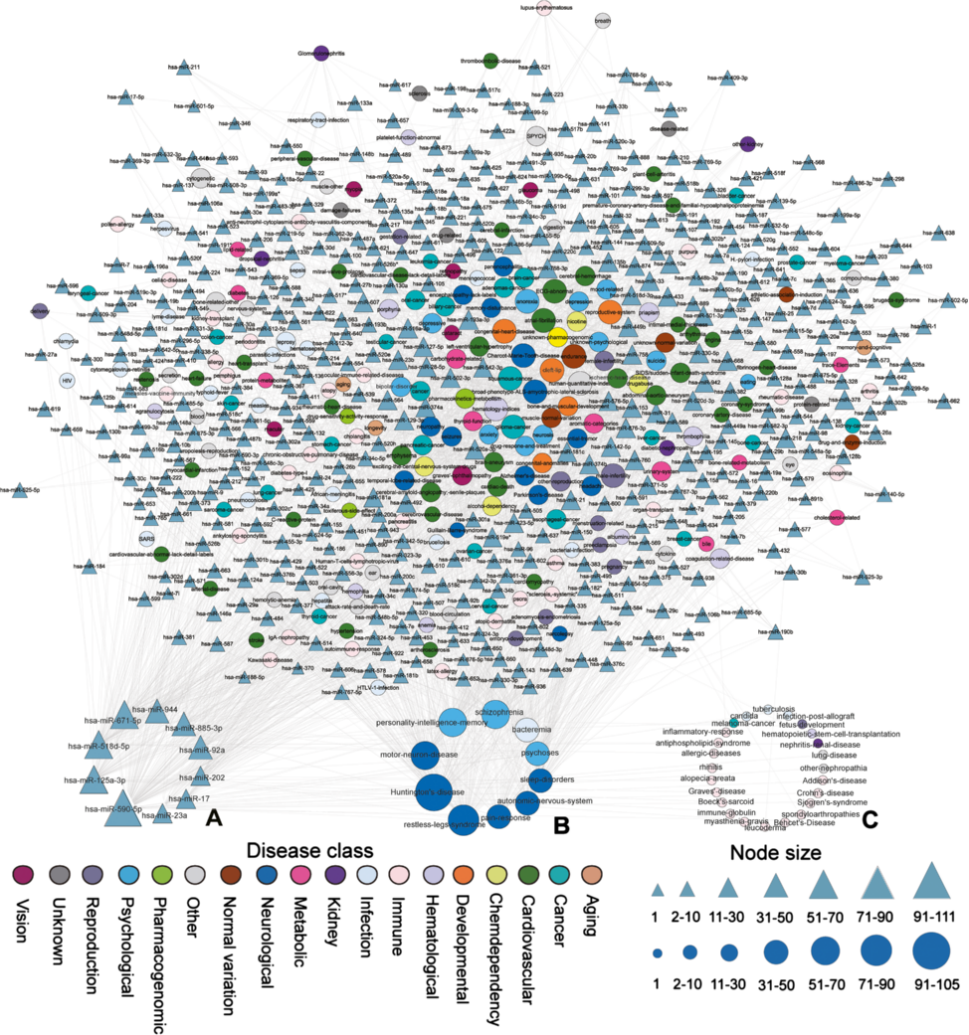
**The constructed miRNA-disease network.** The bipartite network was composed of miRNAs (triangles) and diseases (circles). A disease is linked by miRNA if the p-value is less than 0.05. Disease nodes are colored according to disease class information from GAD; diseases are classified into 18 categories. The size of a node is proportional to the degree of the node, whereas the thickness of an edge is proportional to the p-value; the smaller the p-value the thicker the edge **(A)**. The top 10 largest degree miRNAs in the miRNA-disease network **(B)**. The top 10 largest degree diseases in the miRNA-disease network **(C)**. The diseases associated with only one miRNA in the miRNA-disease network.

We prioritized 499 miRNAs for each of the 269 diseases according to p-values. At a p-value threshold of 0.05, we obtained a miRNA-disease network that included 715 nodes (454 miRNAs and 261 diseases) and 2858 interactions (Figure 2; also see Additional file 2). Squamous cell cancer and glioma cancer were analyzed as two examples (Table 1), and we found that there were eight and six miRNAs in the top 10, respectively. For instance, hsa-miR-183 was ranked at 1 in squamous cell cancer, which has been found to be downregulated in head and neck squamous cell carcinoma by real-time PCR [37]. Hsa-miR-148a, which was ranked at 1 in glioma, was recently determined to be overexpressed in human glioblastoma multiforme by microarray analysis (fold change = 12.030) [38]. These results demonstrated that our method can effectively identify potential miRNA-disease associations, and that we constructed a reliable miRNA-disease network.

**Table 1:** Literature evidence for top 10 miRNAs of squamous cancer and glioma cancer

**Squamous cancer**				**Glioma cancer**			
**miRNA**	**Rank**	**Literature validation**	**PubMed ID**	**miRNA**	**Rank**	**Literature validation**	**PubMed ID**
hsa-miR-183	1	Yes	16192569	hsa-miR-148a	1	Yes	19487573
hsa-miR-573	2	No	-	hsa-miR-148b	2	No	-
hsa-miR-188-5p	3	Yes	16192569	hsa-miR-152	3	Yes	17363563
hsa-miR-34a	4	Yes	18381414	hsa-miR-205	4	No	-
hsa-miR-9	5	Yes	18451220	hsa-miR-20b	5	No	-
hsa-miR-23b	6	Yes	18381414	hsa-miR-589	6	No	-
hsa-miR-518d-3p	7	No	-	hsa-miR-93	7	Yes	19487573
hsa-miR-148b	8	Yes	16192569	hsa-miR-222	8	Yes	19424584
hsa-miR-299-3p	9	Yes	18381414	hsa-miR-130a	9	Yes	16039986
hsa-miR-181d	10	Yes	19351747	hsa-miR-362-3p	10	Yes	19487573

### Global properties of miRNA regulation of disease genes

Next, we analyzed the global properties of miRNA regulation of disease genes by the bipartite miRNA-disease network. Firstly, we investigated the characteristics of miRNAs and diseases in the network based on the degree distribution. We found that the degree distribution for most miRNAs was low, and only a few miRNAs played a global regulatory role in the regulation of a large number of disorders (Additional file 1: Figure S2A). For example, hsa-miR-590-5p exhibited the largest degree and was recently found to be dysregulated in many diseases [39-41]. The top 10 miRNAs that exhibited the largest degree of distribution are shown in Figure 2A. In the other hand, we observed that most of the diseases were associated with only a small number of miRNAs (Additional file 1: Figure S2B). Moreover, some single, complex human diseases were related to numerous miRNAs. Huntington's disease exhibited the largest degree, which is associated with numerous miRNAs such as hsa-miR-128 [42], hsa-miR-9* [43], and hsa-miR-330 [44]. The top 10 diseases exhibiting the largest degree of distribution are shown in Figure 2B.

Secondly, we investigated the correlation between miRNA regulation and disease class. As shown in Additional file 1: Figure S2C and Table 2, we found that neurological diseases exhibited the largest average degree, whereas immune diseases had the smallest average degree. This result indicated that genes associated with neurological diseases tended to be regulated by a higher number of miRNAs. In contrast, genes involved in immune diseases tended to be regulated by fewer miRNAs. This phenomenon is shown in Figure 2C which also illustrates which diseases are associated with only one miRNA. For example, Graves' and Addison's diseases are correlated with only one miRNA and can be regarded as miRNA-specific diseases, which is consistent with the existing knowledge indicating that they are pathway-specific diseases [14].

**Table 2:** The number of diseases and average degree in each disease class

**Disease class**	**Number of diseases**	**Average degree**	**Disease class**	**Number of diseases**	**Average degree**
Neurological	20	33.300	Pharmacogenomic	4	9.250
Developmental	5	27.400	Metabolic	11	8.455
Psychological	14	25.214	Other	21	7.380
Chemdependency	4	20.750	Vision	6	7.167
Normal variation	5	16.400	Kidney	5	6.000
Cancer	28	10.429	Aging	3	5.667
Reproduction	11	10.100	Infection	25	5.360
Hematological	11	9.909	Unknown	5	4.200
Cardiovascular	38	9.316	Immune	45	3.178

To evaluate the effect of the p-value threshold on construction of the miRNA-disease network, another two p-value thresholds, 0.1 and 0.01, were used to analyze certain properties among the miRNA-disease networks. Firstly, we analyzed the correlation of the miRNA degree between each two of the three miRNA-disease networks. As a result, they all significantly positively correlated (see Additional file 1: Table S9). In the same manner, we analyzed the correlation of the disease degree, which yielded similar results (see Additional file 1: Table S9). We also found that the top 10 largest degree of miRNAs and diseases in these three miRNA-disease networks were almost identical (see Additional file 1: Table S10). Secondly, we investigated the correlation between miRNA regulation and disease class in the miRNA-disease networks. The results demonstrated that there was not much change and that the neurological diseases always exhibited the largest average degree (see Additional file 1: Figure S2C and Figure S3).

### MiRNA modules are associated with disease clusters

It has been reported that diseases within the same disease class tend to share a genetic origin and form local functional clustering (modularity) [45]. To explore whether functional clustering existed in our miRNA-disease bipartite network, the diseases in the miRNA-disease network were assigned to 18 disease classes based on GAD. We then used BD and BH measures to quantify the modular properties in the network (for a detailed description, see Additional file 1). Both measures have been used in a previous report to evaluate modularity for bipartite networks [14]. If BD > BH, diseases belonging to the disease class associated with the corresponding miRNAs tend to exhibit clustering phenomena in the network. For cases in which BD > 1 and BH < 1, the diseases within the disease class associated with the corresponding miRNAs exhibit clear clustering tendencies in the network.

**Figure 3:**
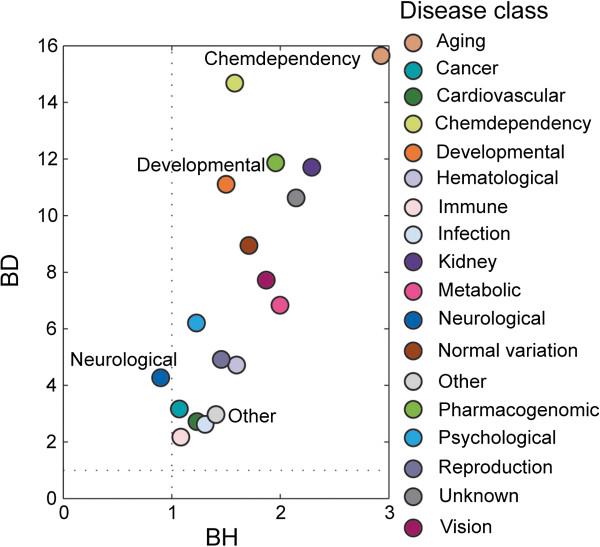
**Using BD and BH for evaluating the clustering phenomenon for each disease class.** If BD > BH, the diseases belonging to the disease class associated with the corresponding miRNAs tend to exhibit clustering phenomena in the network. For cases in which BD > 1 and BH < 1, the diseases within the disease class associated with the corresponding miRNAs exhibit clear clustering tendencies in the network.

We computed the BDs and BHs for the 18 disease classes. As shown in Figure 3, all BDs > 1 and the average value of BDs for these disease classes was up to 7.411, whereas the average value of BHs was low (0.649). For the neurological disease class, we found BD > 1 and BH < 1 (BD = 4.235 and BH = 0.902), suggesting that diseases in this class associated with the corresponding miRNAs display clear functional clustering phenomena. The BDs and BHs of other disease classes all satisfied BD > BH, indicating that diseases in these disease classes associated with the corresponding miRNAs tended to form functional clustering. Interestingly, the developmental disease class (BD/BH = 7.412) and chemical dependency disease class (BD/BH = 8.933) exhibited the largest ratios of BD to BH. However, some disease classes exhibited smaller differences between BD and BH, such as the other disease class that exhibited the smallest ratio (2.074), which was potentially attributable to the overlapping of disorders in other disease classes.

Similarly, we investigated whether the functional clustering of a disease class existed when using different p-value thresholds to construct the miRNA-disease network. For each of the above three miRNA-disease networks, we computed the BDs and BHs. As a result, diseases in the same disease class associated with the corresponding miRNAs displayed functional clustering phenomena in all three networks (see Additional file 1: Table S11), indicating that the results remained stable at different p-value thresholds.

**Figure 4:**
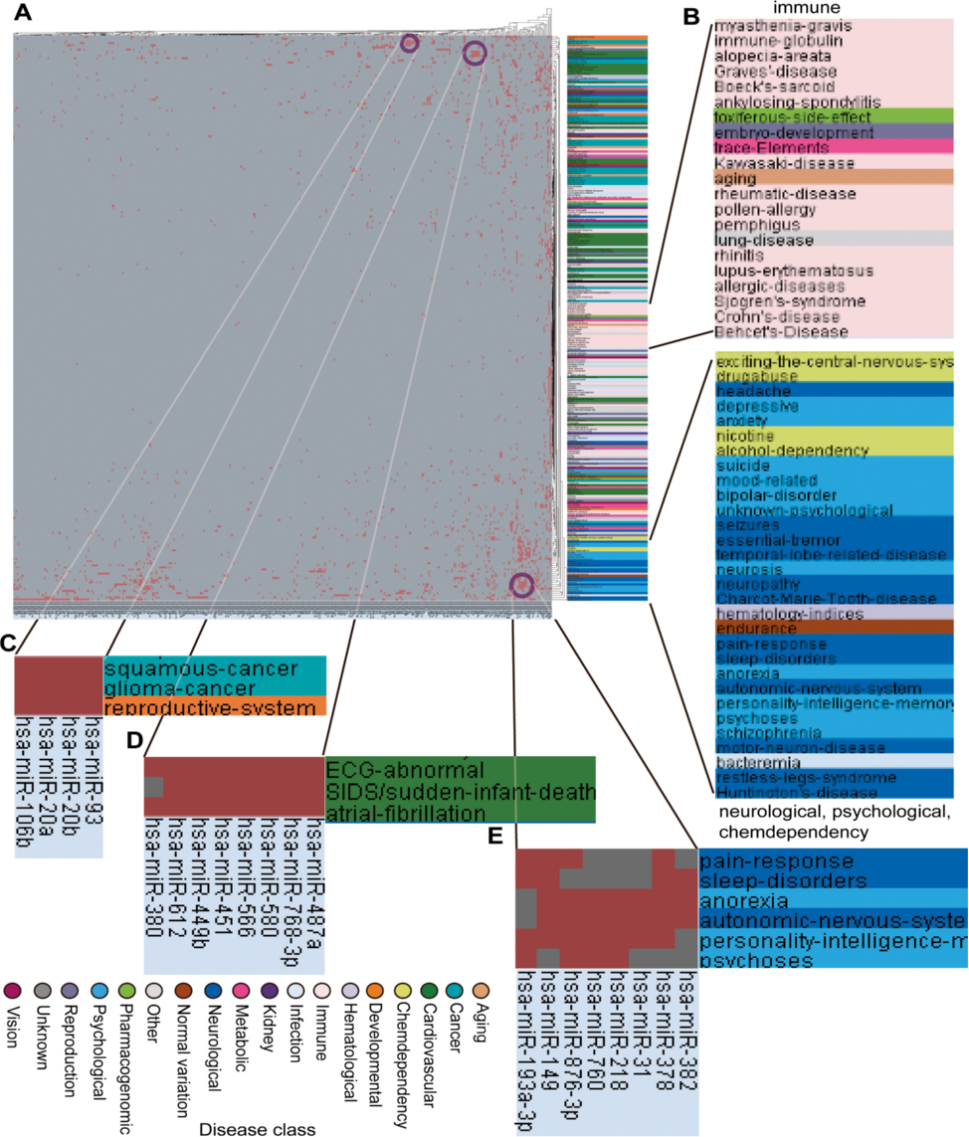
**Hierarchical clustering of the miRNA-disease network. (A)** Hierarchical clustering between 454 miRNAs and 261 diseases. Red cells denote links between the corresponding miRNAs and diseases. Disease labels are colored according to disease class. **(B)** Zoom-in plot of disease labels in Figure 4A. **(C)**, **(D)**, and **(E)** are zoom-in plots of corresponding purple circle regions in Figure 4A.

To further investigate the combinational regulatory effects of miRNAs on disease clusters in the miRNA-disease network, we performed hierarchical clustering on the bipartite network using Cluster3 software by the city-block distance and complete linkage method (shown by JavaTreeView imaging software; Figure 4). The hierarchical clustering method is unsupervised and therefore does not require disease class information for use in our miRNA-disease network to identify miRNA-disease modules. As a result, we found that disorders within the same disease class tended to cluster together (two examples are shown in Figure 4B). Most of the light pink regions that are grouped together denote the immune disease class and most of the dark blue, light blue, and light yellow regions clustered together represent neurological, psychological, and chemical dependency disease classes, respectively. We observed that not all of the disorders in the same disease class gathered into one cluster, and that the cluster included diseases from other classes. This observation may be due to overlapping of different disease classes in which one disease belonging to a disease class is also classified into another disease class. For example, schizophrenia belongs to the psychological disease class (GAD, Dec 15, 2008), but it is also associated with the neurological system (Mesh).

Next, we identified certain co-regulated modules in our miRNA-disease network (Figure 4C–E). As shown in Figure 4C, hsa-miR-93, hsa-miR-20b, hsa-miR-20a, and hsa-miR-106b may jointly regulate genes involved in squamous cancer, glioma cancer, and reproductive system diseases. This finding was in concordance with previous reports showing that the expression of all of these miRNAs is dysregulated in these diseases (for a detailed description, see Additional file 1: Table S12). In addition, all four miRNAs belong to the miR-17 family, and hsa-miR-93 and hsa-miR-106b are located in the same chromosomal region, 7q22.1. MiRNAs of the miR-17 family have been found to regulate cell cycle progression by targeting p21, and contribute to tumorigenesis [46-48]. As shown in Figure 4D, all of the eight miRNAs in this module co-regulated genes involved in the three diseases in the same disease class (cardiovascular disease class), indicating that these diseases might share similar miRNA regulatory mechanisms. Recent findings have provided some evidence in support of this hypothesis. Wang et al. recently reported that loss of the miR-144/miR-451 cluster limits ischemic preconditioning cardio-protection by upregulation of Rac-1-mediated oxidative stress signalling [49]. At the same time, hsa-miR-612 is strongly downregulated (>log2 difference) in differentiated human cardiomyocyte progenitor cells [50]. As illustrated in Figure 4E, all of the eight miRNAs co-regulated genes associated with the six diseases that belonged to the neurological class and psychological class. Psychosis is a psychological disease, but it was also classified as a neurological disorder. We observed that the majority of miRNAs in this module were dysregulated in neurological diseases. For example, hsa-miR-382, hsa-miR-31, and hsa-miR-149 are downregulated in medulloblastoma [51], hsa-miR-378 is downregulated in Alzheimer’s disease [52], and abnormal expression of hsa-miR-218 has been detected in samples from Parkinson’s disease patients [53]. These co-regulated modules may enhance our understanding of the combinational regulatory mechanisms of miRNAs in complex human diseases.

## Discussion

In this study, a computational framework was constructed to identify miRNA-disease associations at the systematic level. The associations were identified based on functional link between miRNA targets and disease genes in PPI network. To search for such functional link, we used a global network distance measure, random walk analysis, which can effectively capture the complex functional associations between miRNA targets and disease genes.

Based on the identified miRNA-disease associations, we constructed a miRNA-disease network to explore the relationships between miRNAs and diseases from a global perspective. In addition, we analyzed the factors that affect the number of diseases associated with miRNAs. We considered two factors for miRNA target genes and the ratios of disease genes to miRNA targets. As a result, the number of diseases linked by miRNA negatively correlated with the number of miRNA targets (*r* = −0.246, *p* = 0.638, Pearson’s correlation; Additional file 1: Figure S4A). The *p* value was not significant, suggesting that there may be no relationship between the number of miRNA targets and the number of associated diseases. We found that the number of diseases linked by miRNA positively correlated with the ratio of disease genes to miRNA targets (*r* = 0.884, *p* = 0.047; Additional file 1: Figure S4B). This result indicated that the more disease genes targeted by a miRNA, the higher the probability that the miRNA is associated with a greater number of diseases.

By analyzing the miRNA-disease bipartite network, we found that diseases in the same disease class tended to cluster together. The hierarchical clustering in this network demonstrated that certain miRNAs combinationally regulated genes involved in a certain type of disease. For future studies, our method can be extended to other kinds of functional modules, such 'as pathway, Gene Ontology, or integrated functional modules, which contain different functional information. This method may be more comprehensive for dissection of the characteristics of miRNA regulation of genes associated with human diseases. Although the results might be affected by different miRNA targets and PPI networks, to make the results more reliable, we collected miRNA targets from seven commonly used miRNA target databases by extracting those with regulatory associations between miRNAs and targets, which appeared in at least three databases. Considering that HPRD included the maximum number of PPIs of any of the publicly available literature-derived databases for human PPIs [54] and the annotations it contained were based on experimental evidence, we chose to compile PPI data from this database. We also used human signaling networks to confirm our approach. With improvements in the quantity and quality of data sources, the miRNA-disease network will be more accurate and comprehensive. In summary, the methods proposed in our study could potentially play an important role in miRNA research and serve as a powerful tool for further elucidation of the molecular basis of human pathologies.

## Conclusions

In conclusion, by focusing on the functional connectivity between miRNA targets and disease genes in PPI network, we developed a computational framework to identify disease-related miRNAs using a global network distance measure realized by RWR algorithm. We further constructed a miRNA-disease network to systematically analyze the global properties of miRNA regulation of disease genes. This will considerably deepen our understanding of the molecular mechanisms of diseases at the post-transcriptional level.

## Competing interests

The authors declare that they have no competing interest.

## Authors’ contributions

XL, ZG and HS conceived and designed the study. HS, LX, CL, LW, ZZ , ZL and WJ collected and integrated the data, analyzed the data and performed the experiments. HS, JX, GZ and XL wrote the paper. All authors read and approved the final manuscript.

## Supplementary Material

Additional file 1 Includes (1) random walk with restart algorithm, (2) obtaining the expression profiles, (3) computation of BD and BH for a disease class in the constructed miRNA-disease network, (4) supplementary Figure S1-S4, and (5) supplementary Table S1-S12. Click here for file

Additional file 2 The miRNA-disease associations. Click here for file
