# Supplementary material for: Walking the interactome to identify human miRNA-disease associations through the functional link between miRNA targets and disease genes
Source: BMC Syst Biol. 2013 Oct 8;7:101. doi: 10.1186/1752-0509-7-101 (PMC4124764; doi:10.1186/1752-0509-7-101)
Supplement: Additional file 1 — Includes (1) random walk with restart algorithm, (2) obtaining the expression profiles, (3) computation of BD and BH for a disease class in the constructed miRNA-disease network, (4) supplementary Figure S1-S4, and (5) supplementary Table S1-S12. [file 1752-0509-7-101-S1.doc]

**This document contains the following Supplementary Text:**

**Random walk with restart algorithm**

**Obtaining the expression profiles**

**Computation of BD and BH for a disease class in the constructed miRNA-disease network**

**This document contains the following Supplementary Figures:**

**Supplementary Figure S1**

**Supplementary Figure S2**

**Supplementary Figure S3**

**Supplementary Figure S4**

**This document contains the following Supplementary Tables:**

**Supplementary Table S1**

**Supplementary Table S2**

**Supplementary Table S3**

**Supplementary Table S4**

**Supplementary Table S5**

**Supplementary Table S6**

**Supplementary Table S7**

**Supplementary Table S8**

**Supplementary Table S9**

**Supplementary Table S10**

**Supplementary Table S11**

**Supplementary Table S12**

**Supplementary Text**

**Random walk with restart algorithm**

We assumed that *G* represents a graph with *N* vertices and *M* edges; the behavior of a random walker was simulated by considering the global structure of a network[1]. The random walker starts from a seed node (or a set of seed nodes, simultaneously) and proceeds to randomly selected neighbors based on edge weights. The aim is to estimate the steady state probability of all of the vertices. As a variant of the random walk, random walk with restart (RWR) is defined as an iterative algorithm. Where $\alpha$ is the probability of the walker returning to the seed node, the closer the value of $\alpha$ is to 0, the more global the view observed. Here, we followed some literatures[2-6] and set $\alpha$ as 0.7. We used *A* to denote the column-normalized adjacency matrix of the graph, and the sum of each column in *A* is one. Formally, the random walk with restart is defined as follows:

$$\text{P}_{t+1}=(1-\alpha)A\text{P}_{t}+\alpha\text{P}_{0}\text{ (1)}$$

where P_0_ is the original probability vector, constructed such that equal probabilities are assigned to all of the seed nodes, with the sum of the probabilities equal to 1. P*_t_* is an *N*-by-1 vector in which the *i*th element represents the probability that the random walker is at node *i* during step *t*. We performed the algorithm until the probability of all of the nodes reached a steady state, measured by the change between P*_t_* and P*_t_*_+1_ (measured by the L_1_ norm) falling below 10^-10^.

The effectiveness of random walk with restart has been demonstrated in previous reports for candidate disease gene prioritization, and it has been shown to outperform many existing network-based gene prioritization algorithms[7, 8]. In this study, we utilized random walk with restart to infer potential miRNA-disease associations.

**Obtaining the expression profiles**

In this study, miRNA expression profiles of nine human cancers were obtained from the Gene Expression Omnibus (GEO) and The Cancer Genome Atlas (TCGA). The expression profiles of prostate cancer, pancreatic cancer, nasopharyngeal cancer and sarcoma cancer were downloaded from GEO (GSE8126, GSE24279, GSE33225 and GSE16353, respectively). The expression profiles of the remaining cancers (breast cancer, glioma cancer, stomach cancer, ovarian cancer and kidney cancer) were downloaded from TCGA. We directly downloaded the normalized miRNA data in each sample from TCGA (level 3 data). For the datasets obtained from GEO, we also downloaded the normalized miRNA expression profiles. Finally, miRNA precursors were mapped to mature miRNAs using miRBase.

**Computation of BD and BH for a disease class in the constructed miRNA-disease network**

Park et al.[9] first proposed D and H to quantify the modular properties for one-mode network (e.g. protein-protein interaction networks), and Li et al.[10] revised this method, BD and BH, for evaluation of modularity for bipartite networks such as the miRNA-disease network. Here, we used BD and BH to investigate the clustering phenomenon in this miRNA-disease bipartite network.

For a given disease class (e.g., the neurological disease class), each disease in the miRNA-disease network was assigned a binary value 1 or 0 with this disease belong to the neurological disease class or not. We let *Dn*_1_ (*Dn*_0_) as the number of disease with the assigned value 1 (0), and the total number of diseases in the miRNA-disease network *Dn* can be computed as *Dn*_1_+ *Dn*_0_. We let *Mn*_1_ as the number of miRNAs associated with neurological disease (means that a miRNA has a link with a disease in the neurological disease class) and *Mn*_0_ as the number of miRNAs associated with diseases not belonging to the neurological disease class, and the total number of miRNAs in the miRNA-disease network *Mn* can be computed as *Mn*_1_+ *Mn*_0_. A disease may belong (1) or not belong (0) to the neurological disease class. A miRNA may belong (1) or not belong (0) to the miRNA associated with neurological diseases. Hence, there exist three types of links between a disease and a miRNA in the network: (1-1), (1-0) and (0-0), and the numbers of them were labeled with *m*­_11_, *m­*_10_ and *m*­_00_ respectively. Thus, the total number of links *M* in the network could be represented as *m*­_11_+*m­*_10_+*m*­_00_.

Suppose each node in the network has an equal chance to belong to the neurological disease class, then the expected values of *m*­_11_ and *m­*_10_ could be calculated as follows:

$$\bar{m}_{11}=\left(\begin{matrix} Dn_{1} \\ 1 \end{matrix}\right)\times\left(\begin{matrix} Mn_{1} \\ 1 \end{matrix}\right)\times p$$

$$\bar{m}_{10}=\left[\left(\begin{matrix} Dn_{1} \\ 1 \end{matrix}\right)\times\left(\begin{matrix} Mn_{0} \\ 1 \end{matrix}\right)+\left(\begin{matrix} Dn_{0} \\ 1 \end{matrix}\right)\times\left(\begin{matrix} Mn_{1} \\ 1 \end{matrix}\right)\right]\times p$$

Where$p=M/(Dn\times Mn)$represents the average probability that a disease is connected with a miRNA in the network. The BD and BH are defined as follows:

$\text{BD}=\frac{m_{11}}{\bar{m}_{11}}$ and $\text{BH}=\frac{m_{10}}{\bar{m}_{10}}$

For each disease class in the miRNA-disease network, BD and BH can be calculated according to the above formula. BD>1 (BD<1) indicates that diseases in the disease class tend to connect more (less) densely with the corresponding miRNAs than expected by chance. Similarly, BH>1 (BH<1) means that diseases in the disease class have more (fewer) connections to miRNAs not belonging to the corresponding miRNAs than the random expectation. If BD>BH, the diseases belonging to the disease class associated with the corresponding miRNAs tend to exhibit clustering phenomena in the network. For cases in which BD>1 and BH<1, the diseases within the disease class associated with the corresponding miRNAs exhibit clear clustering tendencies in the network.

**Supplementary references**

1. Lovász: **Random Walks on Graphs: A Survey.** *Royal Society Mathematical Studies* 1993, **2:**1-46.

2. Chen X, Liu MX, Yan GY: **Drug-target interaction prediction by random walk on the heterogeneous network.** *Mol Biosyst* 2012, **8:**1970-1978.

3. Li Y, Patra JC: **Genome-wide inferring gene-phenotype relationship by walking on the heterogeneous network.** *Bioinformatics* 2010, **26:**1219-1224.

4. Jiang R, Gan M, He P: **Constructing a gene semantic similarity network for the inference of disease genes.** *BMC Syst Biol* 2011, **5 Suppl 2:**S2.

5. Macropol K, Can T, Singh AK: **RRW: repeated random walks on genome-scale protein networks for local cluster discovery.** *BMC Bioinformatics* 2009, **10:**283.

6. Gong NZ, Talwalkar A, Mackey L, Huang L, Shin ECR, Stefanov E, Shi ER, Song D: **Jointly Predicting Links and Inferring Attributes using a Social-Attribute Network (SAN).** *SNA-KDD’12, Aug 12, 2012, Beijing, China* 2012.

7. Kohler S, Bauer S, Horn D, Robinson PN: **Walking the interactome for prioritization of candidate disease genes.** *Am J Hum Genet* 2008, **82:**949-958.

8. Navlakha S, Kingsford C: **The power of protein interaction networks for associating genes with diseases.** *Bioinformatics* 2010, **26:**1057-1063.

9. Park J, Barabasi AL: **Distribution of node characteristics in complex networks.** *Proc Natl Acad Sci U S A* 2007, **104:**17916-17920.

10. Li X, Li C, Shang D, Li J, Han J, Miao Y, Wang Y, Wang Q, Li W, Wu C, et al: **The implications of relationships between human diseases and metabolic subpathways.** *PLoS One* 2011, **6:**e21131.

**Supplementary Figure S1**

**
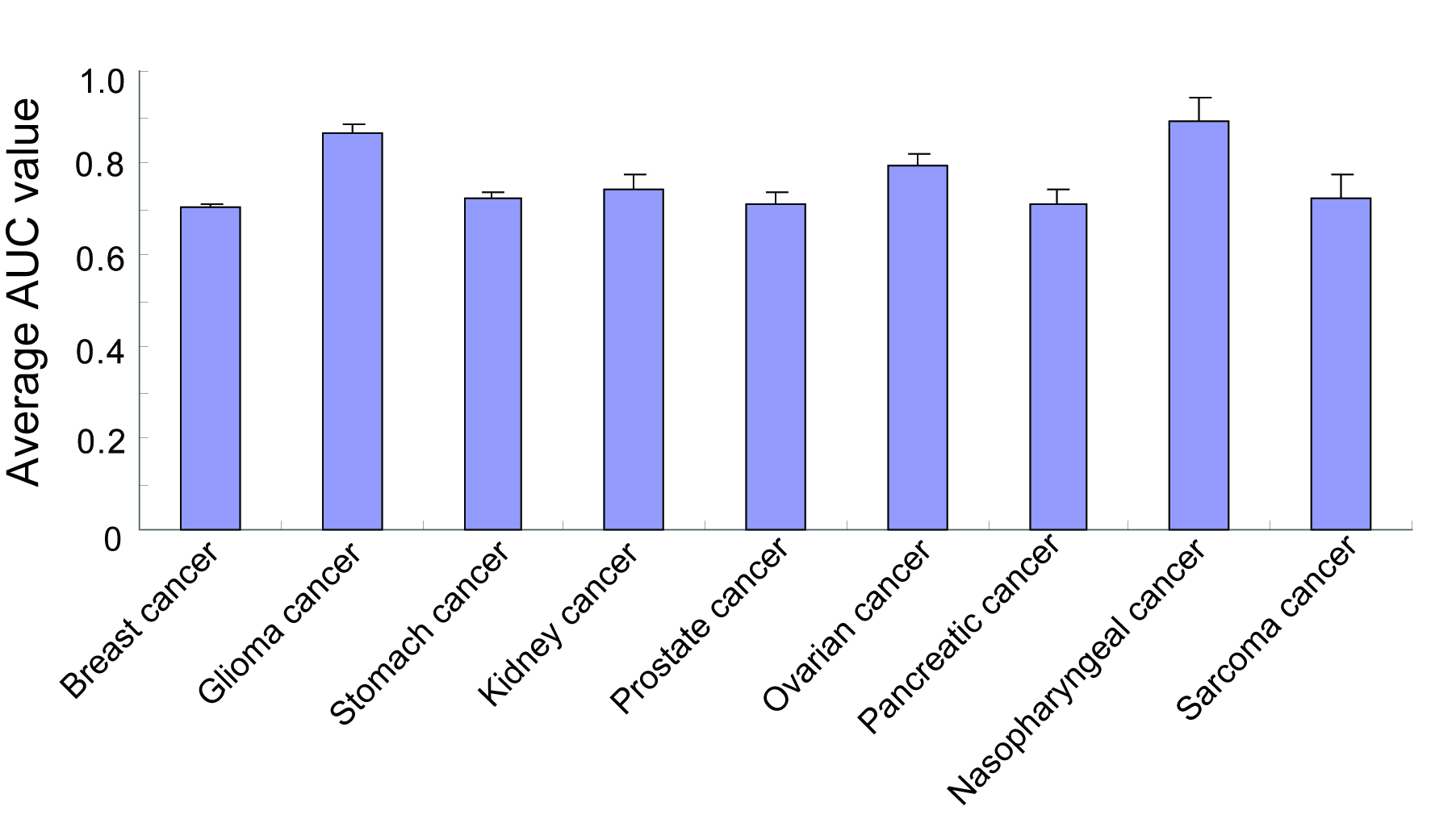
**

**Distribution of average AUC values and standard deviation of nine human cancers with variation of the parameter**$\beta$**.**

**Supplementary Figure S2**


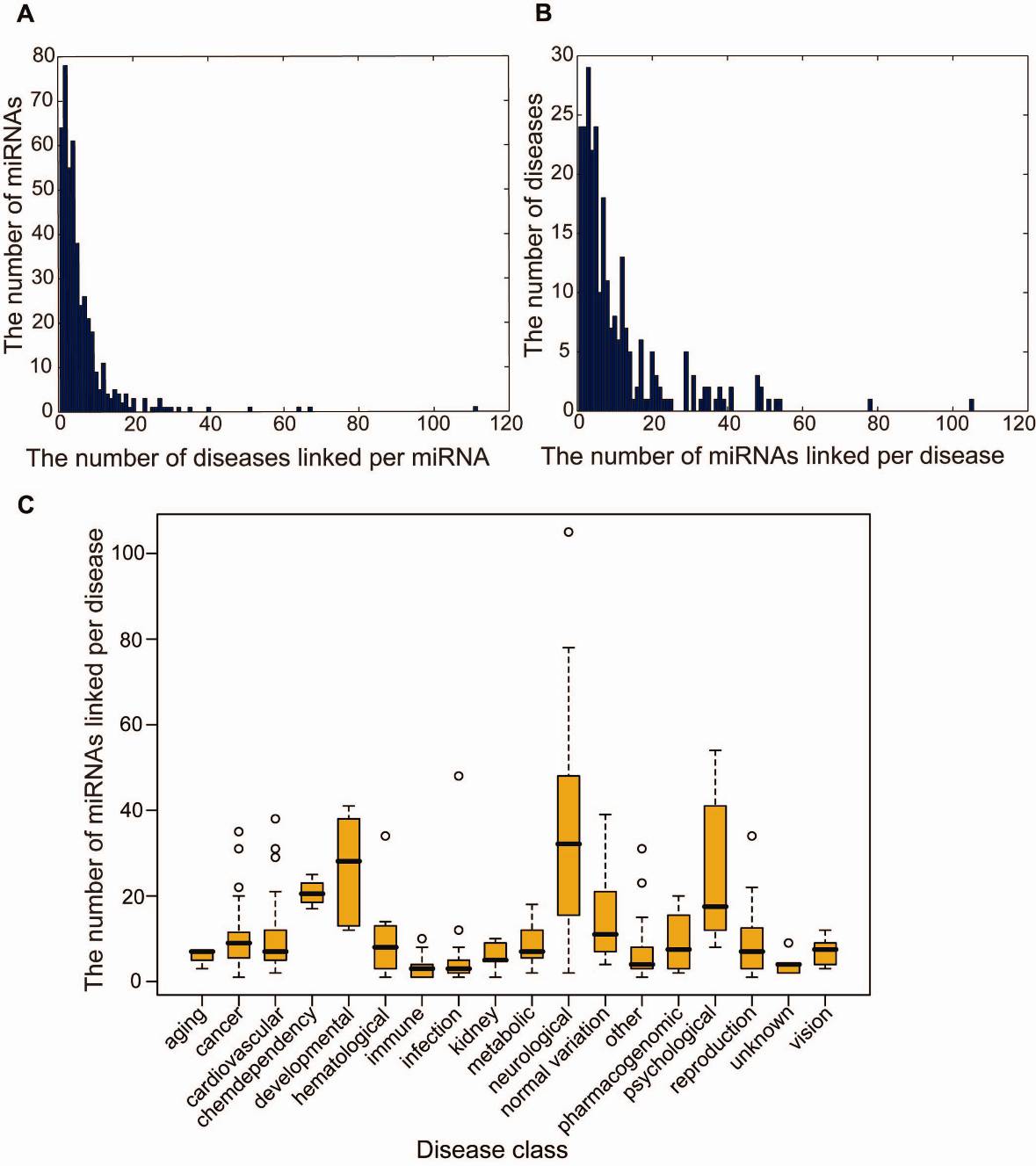


**Distribution of diseases linked per miRNA and disease related miRNAs.** (A) The distribution of the number of diseases linked per miRNA. (B) The distribution of the number of miRNAs linked per disease. (C) The distribution of the number of miRNAs linked per disease class.

**Supplementary Figure S3**


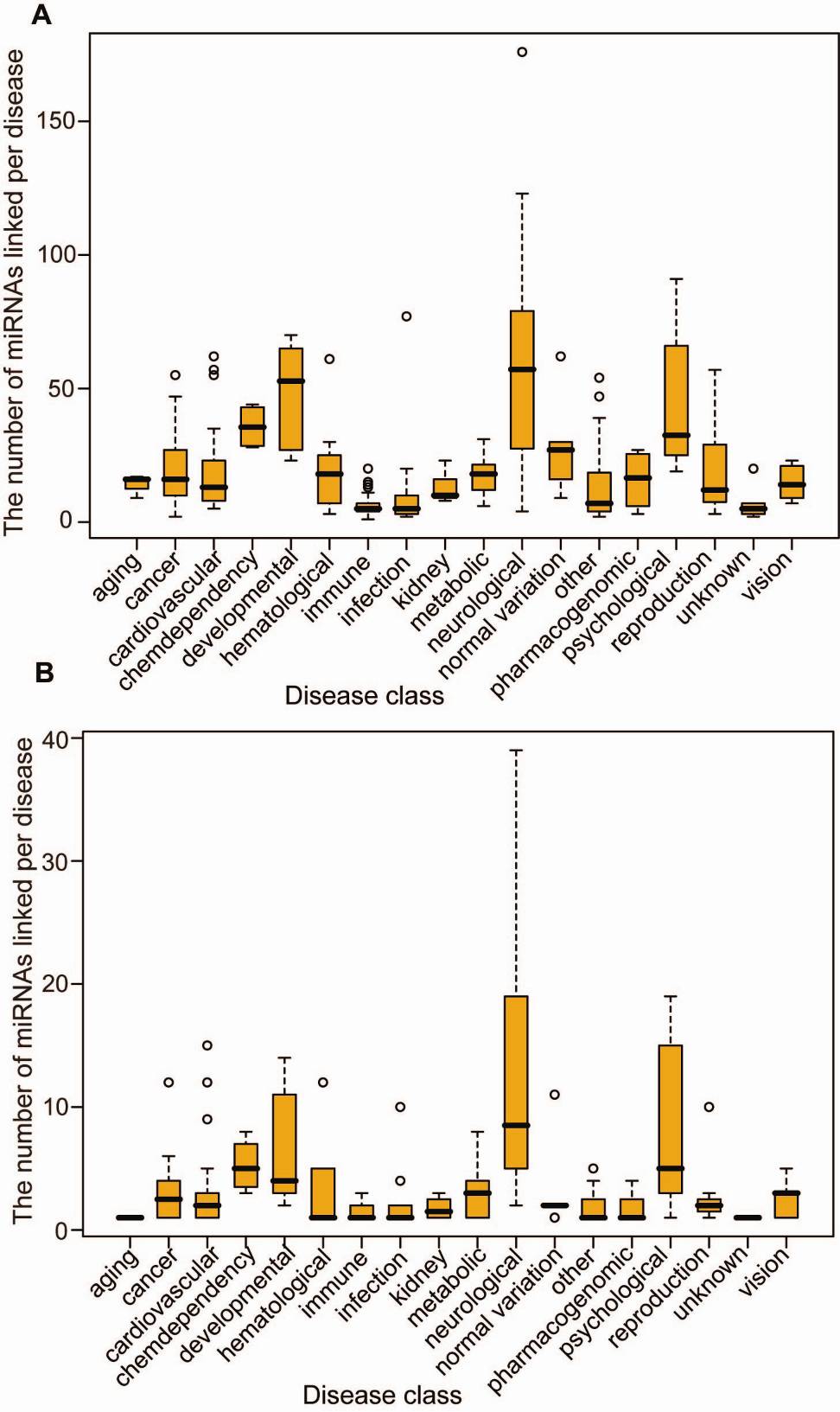


**Distribution of the number of miRNAs linked per disease class in the miRNA-disease networks constructed by different p-value thresholds.** (A) **p-value** threshold selected as 0.1 (B) **p-value** threshold selected as 0.01.

**Supplementary Figure S4**


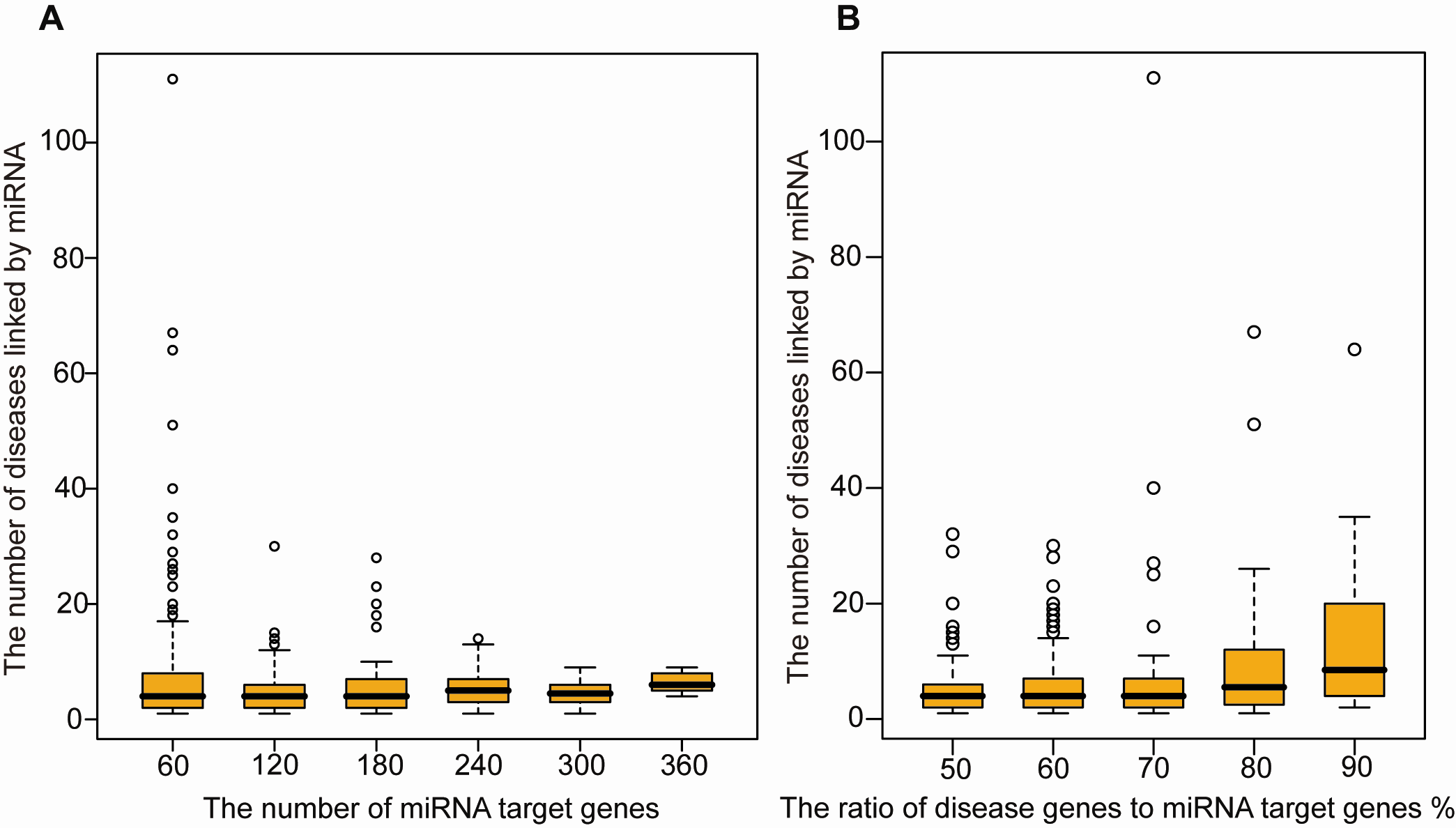


(A) Plot of relationships between the number of miRNA targets and the number of diseases linked by miRNA. (B) The plot of relationships between the ratio of disease genes to miRNA targets and the number of diseases linked by miRNA.

**Supplementary Table S1.** The number of positive miRNA set and negative miRNA set involved in each cancer.

| **Cancer name** | **Breast** | **Glioma** | **Stomach** | **Kidney** | **Prostate** | **Ovarian** | **Pancreatic** | **Nasopharyngeal** | **Sarcoma** |
| --- | --- | --- | --- | --- | --- | --- | --- | --- | --- |
| NO.P | 104 | 39 | 80 | 72 | 65 | 57 | 73 | 19 | 9 |
| NO.N | 104 | 39 | 80 | 72 | 65 | 57 | 73 | 19 | 9 |

**Note**: “NO.P” denotes number of positives; “NO.N” denotes number of negatives.

**Supplementary Table S2.** AUC values of nine human cancers.

| **Cancer**  **name** | **Breast** | **Glioma** | **Stomach** | **Kidney** | **Prostate** | **Ovarian** | **Pancreatic** | **Nasopharyngeal** | **Sarcoma** |
| --- | --- | --- | --- | --- | --- | --- | --- | --- | --- |
| **AUC** | 0.713 | 0.882 | 0.735 | 0.765 | 0.726 | 0.813 | 0.714 | 0.913 | 0.735 |

**Supplementary Table S3.** AUC values of nine human cancers when we used human signaling network.

| **Cancer**  **name** | **Breast** | **Glioma** | **Stomach** | **Kidney** | **Prostate** | **Ovarian** | **Pancreatic** | **Nasopharyngeal** | **Sarcoma** |
| --- | --- | --- | --- | --- | --- | --- | --- | --- | --- |
| **AUC** | 0.682 | 0.809 | 0.752 | 0.749 | 0.734 | 0.842 | 0.720 | 0.953 | 0.833 |

**Supplementary Table S4.** AUC values of four human cancers when we used disease genes identified by DNA sequencing.

| **Cancer**  **name** | **Breast** | **Glioma** | **Ovarian** | **Sarcoma** |
| --- | --- | --- | --- | --- |
| **AUC** | 0.703 | 0.771 | 0.741 | 0.722 |

**Supplementary Table S5.** AUC values of nine human cancers with variation of the parameter$\beta$.

| **Parameter**$\beta$ | **Cancer**  **name** | **AUC** | **Parameter**$\beta$ | **Cancer**  **name** | **AUC** | **Parameter**$\beta$ | **Cancer** | **AUC** |
| --- | --- | --- | --- | --- | --- | --- | --- | --- |
| 0.1 | Breast | 0.696 | 0.1 | Glioma | 0.857 | 0.1 | Stomach | 0.713 |
| 0.2 | Breast | 0.700 | 0.2 | Glioma | 0.872 | 0.2 | Stomach | 0.720 |
| 0.3 | Breast | 0.706 | 0.3 | Glioma | 0.884 | 0.3 | Stomach | 0.732 |
| 0.4 | Breast | 0.710 | 0.4 | Glioma | 0.890 | 0.4 | Stomach | 0.741 |
| 0.5 | Breast | 0.713 | 0.5 | Glioma | 0.882 | 0.5 | Stomach | 0.735 |
| 0.6 | Breast | 0.712 | 0.6 | Glioma | 0.867 | 0.6 | Stomach | 0.734 |
| 0.7 | Breast | 0.705 | 0.7 | Glioma | 0.857 | 0.7 | Stomach | 0.718 |
| 0.8 | Breast | 0.697 | 0.8 | Glioma | 0.849 | 0.8 | Stomach | 0.704 |
| 0.9 | Breast | 0.683 | 0.9 | Glioma | 0.835 | 0.9 | Stomach | 0.684 |
| 0.1 | Kidney | 0.749 | 0.1 | Prostate | 0.713 | 0.1 | Ovarian | 0.806 |
| 0.2 | Kidney | 0.762 | 0.2 | Prostate | 0.719 | 0.2 | Ovarian | 0.812 |
| 0.3 | Kidney | 0.767 | 0.3 | Prostate | 0.722 | 0.3 | Ovarian | 0.815 |
| 0.4 | Kidney | 0.773 | 0.4 | Prostate | 0.733 | 0.4 | Ovarian | 0.817 |
| 0.5 | Kidney | 0.765 | 0.5 | Prostate | 0.726 | 0.5 | Ovarian | 0.813 |
| 0.6 | Kidney | 0.750 | 0.6 | Prostate | 0.726 | 0.6 | Ovarian | 0.790 |
| 0.7 | Kidney | 0.731 | 0.7 | Prostate | 0.699 | 0.7 | Ovarian | 0.772 |
| 0.8 | Kidney | 0.713 | 0.8 | Prostate | 0.681 | 0.8 | Ovarian | 0.751 |
| 0.9 | Kidney | 0.692 | 0.9 | Prostate | 0.642 | 0.9 | Ovarian | 0.738 |
| 0.1 | Pancreatic | 0.736 | 0.1 | Nasopharyngeal | 0.859 | 0.1 | Sarcoma | 0.772 |
| 0.2 | Pancreatic | 0.743 | 0.2 | Nasopharyngeal | 0.888 | 0.2 | Sarcoma | 0.778 |
| 0.3 | Pancreatic | 0.741 | 0.3 | Nasopharyngeal | 0.917 | 0.3 | Sarcoma | 0.765 |
| 0.4 | Pancreatic | 0.732 | 0.4 | Nasopharyngeal | 0.913 | 0.4 | Sarcoma | 0.765 |
| 0.5 | Pancreatic | 0.714 | 0.5 | Nasopharyngeal | 0.913 | 0.5 | Sarcoma | 0.735 |
| 0.6 | Pancreatic | 0.706 | 0.6 | Nasopharyngeal | 0.953 | 0.6 | Sarcoma | 0.685 |
| 0.7 | Pancreatic | 0.687 | 0.7 | Nasopharyngeal | 0.932 | 0.7 | Sarcoma | 0.679 |
| 0.8 | Pancreatic | 0.667 | 0.8 | Nasopharyngeal | 0.882 | 0.8 | Sarcoma | 0.667 |
| 0.9 | Pancreatic | 0.649 | 0.9 | Nasopharyngeal | 0.776 | 0.9 | Sarcoma | 0.630 |

**Supplementary Table S6.** AUC values of nine human cancers with variation of the parameter$\alpha$.

| **Parameter** $\alpha$ | **Breast** | **Glioma** | **Stomach** | **Kidney** | **Prostate** | **Ovarian** | **Pancreatic** | **Nasopharyngeal** | **Sarcoma** |
| --- | --- | --- | --- | --- | --- | --- | --- | --- | --- |
| 0.1 | 0.661 | 0.714 | 0.650 | 0.615 | 0.678 | 0.704 | 0.666 | 0.742 | 0.600 |
| 0.3 | 0.693 | 0.842 | 0.677 | 0.669 | 0.713 | 0.799 | 0.740 | 0.812 | 0.667 |
| 0.5 | 0.707 | 0.867 | 0.709 | 0.698 | 0.733 | 0.823 | 0.753 | 0.820 | 0.704 |
| 0.7 | 0.713 | 0.882 | 0.735 | 0.765 | 0.726 | 0.813 | 0.714 | 0.913 | 0.735 |
| 0.9 | 0.713 | 0.843 | 0.711 | 0.747 | 0.690 | 0.780 | 0.661 | 0.921 | 0.790 |

**Supplementary Table S7.** AUC values of nine human cancers when we performed 5000 random PPI networks.

| **Cancer**  **name** | **Breast** | **Glioma** | **Stomach** | **Kidney** | **Prostate** | **Ovarian** | **Pancreatic** | **Nasopharyngeal** | **Sarcoma** |
| --- | --- | --- | --- | --- | --- | --- | --- | --- | --- |
| **AUC** | 0.710 | 0.888 | 0.735 | 0.768 | 0.741 | 0.818 | 0.724 | 0.945 | 0.716 |

**Supplementary Table S8.** AUC values of nine human cancers when we utilized hypergeometric distribution method.

| **Cancer**  **name** | **Breast** | **Glioma** | **Stomach** | **Kidney** | **Prostate** | **Ovarian** | **Pancreatic** | **Nasopharyngeal** | **Sarcoma** |
| --- | --- | --- | --- | --- | --- | --- | --- | --- | --- |
| **AUC** | 0.673 | 0.649 | 0.645 | 0.736 | 0.663 | 0.676 | 0.703 | 0.743 | 0.674 |

**Supplementary Table S9.** The Pearson’s correlation coefficients of miRNA degree in the miRNA-disease networks constructed at different p-value thresholds and the Pearson’s correlation coefficients of disease degree in the miRNA-disease networks constructed at different p-value thresholds.

| **miRNA** | | | **Disease** | | |
| --- | --- | --- | --- | --- | --- |
| **(0.1, 0.05)** | **(0.1, 0.01)** | **(0.05, 0.01)** | **(0.1, 0.05)** | **(0.1, 0.01)** | **(0.05, 0.01)** |
| *r*= 0.959,  p<10^-32^ | *r*= 0.822  p<10^-32^ | *r*= 0.898,  p<10^-32^ | *r*= 0.983,  p<10^-32^ | *r*= 0.885,  p<10^-32^ | *r*= 0.914,  p<10^-32^ |

**Supplementary Table S10.** The top 10 largest degree of miRNAs and diseases in the miRNA-disease networks constructed at different p-value thresholds.

| **miRNA** | | | **Disease** | | |
| --- | --- | --- | --- | --- | --- |
| **p-value<0.1** | **p-value<0.05** | **p-value <0.01** | **p-value <0.1** | **p-value <0.05** | **p-value <0.01** |
| hsa-miR-590-5p | hsa-miR-590-5p | hsa-miR-590-5p | Huntington's-disease | Huntington's-disease | Huntington's-disease |
| hsa-miR-125a-3p | hsa-miR-125a-3p | hsa-miR-125a-3p | restless-legs-syndrome | restless-legs-syndrome | restless-legs-syndrome |
| hsa-miR-518d-5p | hsa-miR-518d-5p | hsa-miR-518d-5p | schizophrenia | personality-intelligence-memory | motor-neuron-disease |
| hsa-miR-944 | hsa-miR-671-5p | hsa-miR-218 | sleep-disorders | schizophrenia | autonomic-nervous-sys |
| hsa-miR-671-5p | hsa-miR-944 | hsa-miR-944 | motor-neuron-disease | motor-neuron-disease | schizophrenia |
| hsa-miR-516a-3p | hsa-miR-885-3p | hsa-miR-202 | personality-intelligence-memory | sleep-disorders | sleep-disorders |
| hsa-miR-92a | hsa-miR-92a | hsa-miR-671-5p | pain-response | psychoses | personality-intelligence-memory |
| hsa-miR-885-3p | hsa-miR-202 | hsa-miR-193a-3p | autonomic-nervous-system | bacteremia | psychoses |
| hsa-miR-668 | hsa-miR-17 | hsa-miR-23a | bacteremia | pain-response | atrial-fibrillation |
| hsa-miR-202 | hsa-miR-23a | hsa-miR-760 | psychoses | autonomic-nervous-system | anorexia |

**Supplementary Table S11.** The BD and BH values of 18 disease classes in the miRNA-disease networks constructed at different p-value thresholds.

| **p-value<0.1** | | | **p-value<0.05** | | | **p-value<0.01** | | |
| --- | --- | --- | --- | --- | --- | --- | --- | --- |
| **Disease class** | **BD** | **BH** | **Disease class** | **BD** | **BH** | **Disease class** | **BD** | **BH** |
| aging | 9.040 | 2.019 | aging | 15.663 | 2.950 | aging | 25.113 | 4.333 |
| cancer | 2.291 | 1.116 | cancer | 3.179 | 1.084 | cancer | 4.637 | 0.535 |
| cardiovascular | 1.826 | 1.209 | cardiovascular | 2.806 | 1.222 | cardiovascular | 4.346 | 1.008 |
| chemdependency | 9.137 | 1.578 | chemdependency | 14.338 | 1.605 | chemdependency | 28.252 | 1.126 |
| developmental | 7.146 | 1.354 | developmental | 11.248 | 1.518 | developmental | 15.524 | 1.862 |
| hematological | 3.254 | 1.553 | hematological | 4.777 | 1.620 | hematological | 9.659 | 0.783 |
| immune | 1.469 | 1.200 | immune | 2.345 | 1.096 | immune | 6.564 | 0.890 |
| infection | 1.777 | 1.270 | infection | 2.737 | 1.256 | infection | 6.849 | 1.174 |
| kidney | 6.351 | 2.072 | kidney | 11.846 | 2.392 | kidney | 26.368 | 3.733 |
| metabolic | 4.058 | 1.749 | metabolic | 6.491 | 2.013 | metabolic | 11.772 | 2.270 |
| neurological | 3.351 | 0.895 | neurological | 4.235 | 0.902 | neurological | 5.720 | 0.883 |
| normal variation | 6.254 | 1.540 | normal variation | 9.189 | 1.754 | normal variation | 15.068 | 1.025 |
| other | 1.851 | 1.432 | other | 2.884 | 1.391 | other | 6.936 | 1.556 |
| pharmacogenomic | 7.292 | 1.953 | pharmacogenomic | 11.985 | 2.087 | pharmacogenomic | 25.113 | 1.501 |
| Psychological | 4.292 | 1.160 | Psychological | 6.078 | 1.195 | Psychological | 7.888 | 0.704 |
| reproduction | 3.086 | 1.366 | reproduction | 4.891 | 1.439 | reproduction | 10.763 | 1.862 |
| unknown | 5.205 | 1.946 | unknown | 10.883 | 2.249 | unknown | 37.669 | 5.173 |
| vision | 5.216 | 1.786 | vision | 7.819 | 1.909 | vision | 15.068 | 1.283 |

Note: If BD>BH, the diseases belonging to the disease class associated with the corresponding miRNAs tend to exhibit clustering phenomena in the network. For cases in which BD>1 and BH<1, the diseases within the disease class associated with the corresponding miRNAs exhibit clear clustering tendencies in the network.

**Supplementary Table S12.** Detailed description of confirmed relationships between miRNAs and diseases in Figure 4C.

| **miRNA** | **Disease** | **Expression pattern of miRNA** | **Detailed description** | **Reference/PubMed ID** | **Year** |
| --- | --- | --- | --- | --- | --- |
| Hsa-miR-93 | Head and neck squamous cell carcinomas | Up-regulated | MiR-106b-25 cluster could be activated by E2F1, in parallel with its host gene Mcm7, which in turn interferes with TGF-β signaling via suppression of p21 mediated by miR-106b and miR-93 as well as silencing of Bim via miR-25. As an added level of complexity, miR-106b and miR-93 can independently regulate E2F1 expression, leading to a negative feedback loop, which is probably important in preventing E2F1 self-activation and apoptosis | Comprehensive microRNA profiling for head and neck  Squamous cell carcinomas/ 20145181 | 2010 |
|  | Glioblastoma multiforme | Up-regulated | Hsa-miR-93 exhibited 2.86-fold higher expression in at least two of three sampled glioblastoma multiformes relative to two normal control brains. | The PTEN-regulating microRNA miR-26a  is amplified in high-grade glioma  and facilitates gliomagenesis in vivo/ 17487573 | 2009 |
|  | [Ovarian cancer](http://www.ncbi.nlm.nih.gov/entrez/dispomim.cgi?id=604370) | Up-regulated | Hsa-miR-93 differentially expressed with >2-fold change in tumor versus normal ovarian tissues in at least 12 of 20 ovarian cancer patient. Higher expression of miR-93 was significantly correlated with a poor prognosis (P < 0.05) in patients with serous ovarian carcinoma. | MicroRNA expression profiles in serous ovarian carcinoma/ 18451233 | 2008 |
| Hsa-miR-20a | Head and neck squamous cell carcinomas | Up-regulated | Hsa-miR-20a differentially expressed between head and neck squamous cell carcinoma and normal tissues. Hsa-miR-20a located in the miR-17-92 cistron at the c13ORF25 of 13q31 and this region is frequently amplified in HNSCC | Comprehensive microRNA profiling for head and neck  Squamous cell carcinomas/ 20145181 | 2010 |
|  | Glioma cancer | Up-regulated | The glioma tissues showed significantly up-regulated expression of miR-20a compared with normal brain tissues (P=0.035). The expression level of miR-20a was higher in high-grade than in low-grade gliomas. miR-20a mimics significantly enhanced the proliferation of U251 cells and the percentage of S-phase cells. miR-20a shows potent effect in promoting the growth of glioma cells, suggesting its important role in the pathogenesis of human glioma. | Expression of hsa-miR-20a in human glioma tissues and its effect on the proliferation of human glioma cells in vitro/ 22381757 | 2012 |
|  | [Ovarian cancer](http://www.ncbi.nlm.nih.gov/entrez/dispomim.cgi?id=604370) | Up-regulated | Hsa-miR-20a differentially expressed with >2-fold change in tumor versus normal ovarian tissues in at least 12 of 20 ovarian cancer patient. | MicroRNA expression profiles in serous ovarian carcinoma/ 18451233 | 2008 |
| Hsa-miR-20b | Head and neck squamous cell carcinomas | Up-regulated | Hsa-miR-20b differentially expressed between head and neck squamous cell carcinoma and normal tissues. miR-20b which belong to the homologue of miR-106a-92 cluster, located on chromosome  Xq26. this region is frequently amplified in HNSCC | Comprehensive microRNA profiling for head and neck  Squamous cell carcinomas/ 20145181 | 2010 |
|  | Hlioma cancer | Unknown | Polyunsaturated fatty acids (PUFAs) can be used to treat glioma cells, hsa-miR-20b differentially expressed in response to PUFA treatment and was induced by PUFA treatments. Most of the targets of hsa-miR-20b were apoptotic genes. | MicroRNA profile of polyunsaturated fatty acid treated glioma cells reveal apoptosis-specific expression changes/ 21961478 | 2011 |
|  | [Ovarian cancer](http://www.ncbi.nlm.nih.gov/entrez/dispomim.cgi?id=604370) | Down-regulated | Hsa-miR-20b was down-regulated, which is  validated by qRT-PCR. hsa-miR-20b targets highest number of genes in MAPK signaling pathway (32 genes) which includes FAS ligand, FGF4, TGF-b receptor 2 (TGFbR2), and various MAP Kinases. Activation of MAPK via phosphorylation can lead to either cell proliferation or apoptosis. | MicroRNA signature of cis-platin resistant vs. cis-platin sensitive ovarian cancer cell lines/21939554 | 2011 |
| Hsa-miR-106b | Head and neck squamous cell carcinomas | Up-regulated | miR-106b-25 cluster could be activated by E2F1, in parallel with its host gene Mcm7, which in turn interferes with TGF-β signaling via suppression of p21 mediated by miR-106b and miR-93 as well as silencing of Bim via miR-25. As an added level of complexity, miR-106b and miR- 93 can independently regulate E2F1 expression, leading to a negative feedback loop, which is probably important in preventing E2F1 self-activation and apoptosis | Comprehensive microRNA profiling for head and neck  Squamous cell carcinomas/ 20145181 | 2010 |
|  | Glioblastoma multiforme | Up-regulated | Hsa-miR-106b exhibited 2.49-fold higher expression in at least two of three sampled glioblastoma multiformes relative to two normal control brains. | The PTEN-regulating microRNA miR-26a  is amplified in high-grade glioma  and facilitates gliomagenesis in vivo/ 17487573 | 2009 |
|  | [Ovarian cancer](http://www.ncbi.nlm.nih.gov/entrez/dispomim.cgi?id=604370) | Up-regulated | Hsa-miR-106b exhibited 3.97-fold higher expression in recurrent versus primary ovarian cancers. | Potential role of miR-9 and miR-223 in recurrent ovarian cancer/ 18442408 | 2008 |
